# CMA analysis identifies homozygous deletion of *MCPH1* in 2 brothers with primary Microcephaly-1

**DOI:** 10.1186/s13039-017-0334-4

**Published:** 2017-09-05

**Authors:** Morteza Hemmat, Melissa J Rumple, Loretta W Mahon, Melanie Morrow, Tamara Zach, Arturo Anguiano, Mohamed M Elnaggar, Boris T Wang, Fatih Z Boyar

**Affiliations:** 1 Quest Diagnostics Nichols Institute, 33608 Ortega Highway, San Juan Capistrano, CA USA; 2 Banner Child Neurology, 5310 W Thunderbird Rd, Ste 301, Glendale, AZ 85306 USA

**Keywords:** Primary microcephaly, *MCPH1* gene, Microdeletion

## Abstract

**Background:**

Homozygous mutations and deletions of the microcephalin gene (*MCPH1*; OMIM *607117) have been identified as a cause of autosomal recessive primary microcephaly and intellectual disability (MIM #251200). Previous studies in families of Asian descent suggest that the severity of the phenotype may vary based on the extent of the genomic alteration. We report chromosome microarray (CMA) findings and the first described family study of a patient with primary microcephaly in a consanguineous Hispanic family.

**Case presentation:**

The proband, a boy born at full-term to consanguineous parents from Mexico, presented at 35 months of age with microcephaly, abnormal brain MRI findings, underdeveloped right lung, almond-shaped eyes, epicanthal folds, bilateral esotropia, low hairline, large ears, smooth philtrum, thin upper lip, and developmental delay. MRI of the brain showed a small dermoid or lipoma (without mass effect) within the interpeduncular cistern and prominent arachnoid granulation. The underdeveloped right lung was managed with long-acting inhaled corticosteroids. Otherwise the proband did not have any other significant medical history. The proband had 2 older brothers, ages 14 and 16, from the same consanguineous parents. The 14-year-old brother had a phenotype similar to that of the proband, while both parents and the oldest brother did not have the same phenotypic findings as the proband. The SNP-based CMA analysis of the proband detected a homozygous 250-kb microdeletion at 8p23.2p23.1, extending from 6,061,169 to 6,310,738 bp [hg19]. This genomic alteration encompasses the first 8 exons of *MCPH1*. Follow-up studies detected the same homozygous deletion in the affected brother, segregating with microcephaly and intellectual disability. Regions of homozygosity (ROHs) were also observed in the affected brother. Since ROHs are associated with an increased risk for recessive disorders, presence of ROH may also contribute to the phenotype of the affected brothers. The parents were both hemizygous for the deletion.

**Conclusion:**

Here we report a homozygous deletion of multiple exons of the *MCPH1* gene that was associated with primary microcephaly and intellectual disability in a Hispanic family. In the context of previous studies, our results support the idea that deletions involving multiple exons cause a more severe phenotype than point mutations.

## Background

Primary microcephaly (MCPH) is a genetically heterogeneous disorder, predominately showing an autosomal recessive mode of inheritance. MCPH is a developmental defect of the brain characterized by a congenital small cranium with a reduced occipito-frontal head circumference (OFC) of more than 2 standard deviations (SD) below the mean for age, sex, and ethnicity (severe microcephaly OFC < 3 SD). MCPH is also characterized by mild to moderate intellectual disability and mild seizures, simplified gyral pattern, periventricular neuronal heterotopias, polymicrogyria, speech delay, hyperactivity and attention deficit, aggressiveness, focal or generalized seizures, and delay of developmental milestones and pyramidal signs [1, 2].

Mutations in 18 genes are known to cause MCPH; *MCPH1* (*607117) was the first gene to be identified [3, 4]. Whole exome (WES) or the whole genome (WGS) studies revealed an additional 17 OMIM genes associated with MCPH including *WDR62* (*613583), *CDK5RAP2* (*608201), *CASC5* (*609173), *ASPM* (*605481), *CENPJ* (*609279), *STIL* (*181590), *CEP135* (*611423), *CEP152* (*613529), *ZNF335* (*610827), *PHC1* (*602978), *CDK6* (*603368), *CENPE* (*117143), *CENPF* (*600235), *PLK4* (*605031), *TUBGCP6* (*610053), *CEP63* (*614724), *NDE1* (*609449) [5].

MCPH caused by *MCPH1* mutation presents with congenital microcephaly, intellectual disability, and a head circumference that is reduced to a greater degree than height [6].

The *MCPH1*/microcephalin gene (*607117) is located at chromosome 8p23 and has a genomic size of 241,905 bp. The open reading frame is 8032 bp and consists of 14 exons that encode 835 amino acids; 3 isoforms have been reported so far [4]. Microcephalin, the encoded protein, is implicated in chromosome condensation and cellular responses that are induced by DNA damage [7]. This protein is thought to have a role in G2/M checkpoint arrest via maintenance of inhibitory phosphorylation of cyclin-dependent kinase 1 [7–9]. Loss of the microcephalin protein thus triggers early mitotic entry of neuroprogenitor cells leading to the inability to maintain brain size [7–9].

Two types of mutations in *MCPH1* have been reported in the literature. A missense mutation was identified in patients with a less severe cellular phenotype and mild microcephaly [6, 10, 11]. Deletions in *MCPH1* have also been reported. A deletion of the first 6 exons of the gene was identified in an Iranian family with intellectual disability and mild microcephaly; premature chromosome condensation in at least 10% to 15% of cells was also reported for this family [11]. In addition, a deletion of the first 11 exons was identified in an Asian-Indian patient [12].

Here we present a consanguineous Hispanic family with primary microcephaly and intellectual disabilities associated with *MCPH1* deletions.

## Case presentation

The proband presented at 35 months of age with microcephaly (44 cm head circumference, which is more than 2 SD below the third percentile for age), dysmorphic facial features (almond shaped eyes, epicanthal folds, bilateral esotropia, low hairline, large ears, smooth philtrum, and thin upper lip), and intellectual disability or developmental delay.

**Fig. 1:**
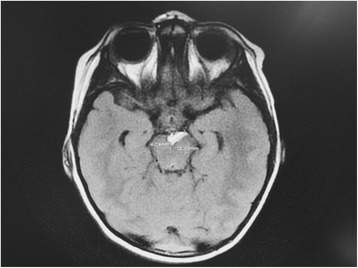
MRI image from the proband. MRI finding indicated a small dermoid or lipoma within the interpeduncular cistern without mass effect and prominent arachnoid granulation

The proband’s MRI of the brain showed a small dermoid or lipoma (without mass effect) within the interpeduncular cistern and prominent (1.2 × 0.4 cm) arachnoid granulation (Fig. 1). The proband also had an underdeveloped right lung, which was managed with long-acting inhaled corticosteroids. Otherwise the proband did not have any other significant medical history. The proband was born via full-term vaginal delivery at 2720 g. The 34-year old mother was G4P3A1L3 and had no known maternal complications or infections. The proband had no prenatal, perinatal, or postnatal complications, and he was discharged on day 2 of life. He did not walk until he was 13 months and did not speak words until 2 years old, so he received physical and speech therapy for diagnosis of developmental delay. At 48 months, <25% of his speech was intelligible to strangers, and he did not form sentences.

**Fig. 2:**
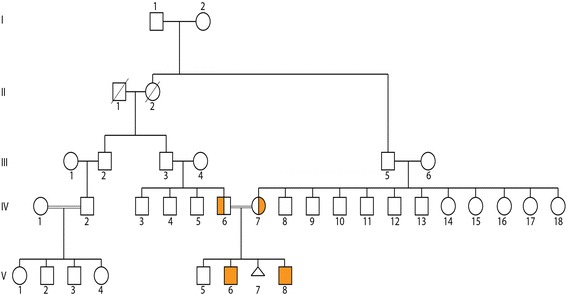
Pedigree of the proband. The proband (V.8) has 2 brothers, V.5 (normal) and V.6 (affected) from the same consanguineous parents (IV.6 an IV.7)

The proband had two brothers who were 14 and 16 years old and from the same consanguineous parents (Fig. 2). One of the brothers (V.6) presented with a similar phenotype as the proband (V.8), while both parents were phenotypically normal (IV.6 and IV.7). The pedigree indicated consanguinity in offspring who had microcephaly, intellectual disabilities, ptosis, hearing loss, and short stature. Hypertension, diabetes, high cholesterol, arthritis, stroke, and osteoporosis were also present in these offspring.

The consanguineous parents of the proband had normal head circumferences for their ages (57 cm for the father, 57.5 cm for the mother). They did not finish high school and stated it was due to marriage at young age. Their country of origin was Mexico. There was no history of seizures or cardiac, renal, skeletal, or metabolic disease noted in the proband or any of his first degree relatives. In order to determine the cause of the patient’s abnormality, CMA was ordered for both parents.

## Methods

Genomic DNA was extracted from whole blood using the Gentra Puregene kit (Qiagen-Sciences, Maryland, USA). Microdeletion/microduplication screening was performed for the proband, his parents, and available brothers using a SNP-array platform (CytoScan HD; Affymetrix, Santa Clara, CA), following the manufacturer’s instruction. The CytoScan HD array has 2.67 million probes, including 1.9 million copy number probes and 0.75 million SNP probes. Array data were analyzed using the Chromosome Analysis Suite (ChAS) (Affymetrix, Inc.) software v2.0. FISH analysis using BlueGnome probes RP11-11I1 (Illumina, San Diego, CA, USA) for the deleted region 8p23.2p23.1 was performed on metaphase cells according to the manufacturer’s protocol. Subsequently, 50 cells were examined carefully.

## Results and discussion

**Fig. 3:**
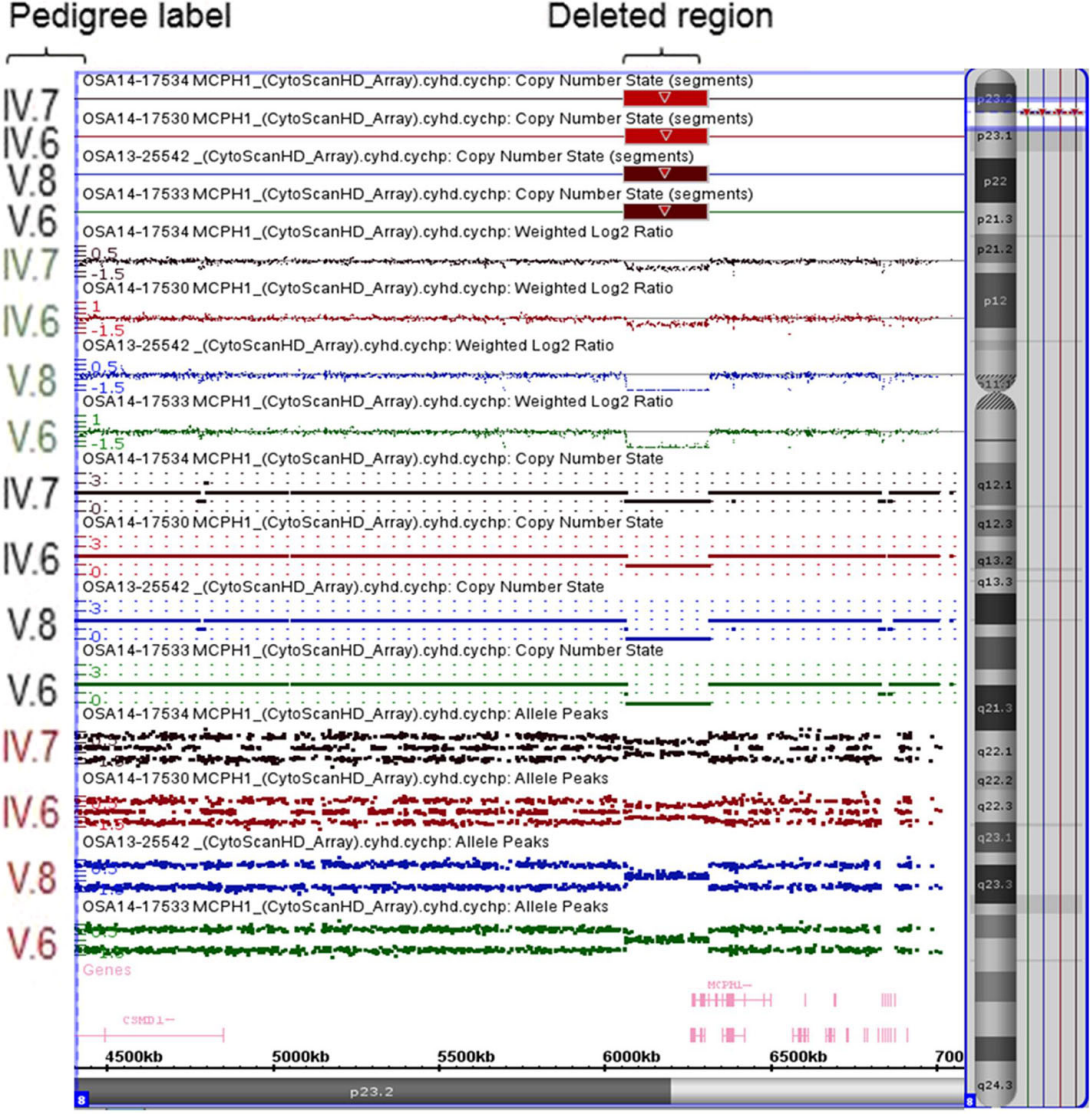
Chromosome microarray analysis. Chromosome 8 with deletion at 8p23.2p23.1 is shown along the right side of the image. SNP-array results show the copy number state (black pedigree labels), weighted log2 ratio (green pedigree labels), and allele peaks (red pedigree labels) for proband (V.8), his brother (V.6), his mother (IV.6), his father (IV.7), and the *MCPH1* gene at the deleted region

The SNP-microarray analysis of the proband’s DNA identified a homozygous microdeletion of 250 kb at 8p23.2p23.1 (Fig. 3). The deletion extended from 6,061,169 to 6,310,738 bp (UCSC genome Browser; http://genome.ucsc.edu/; hg19 release). This genomic alteration indicated a homozygous loss (zero copy) of the first 8 exons of the microcephalin gene (*MCPH1*; OMIM #607117).

**Fig. 4:**
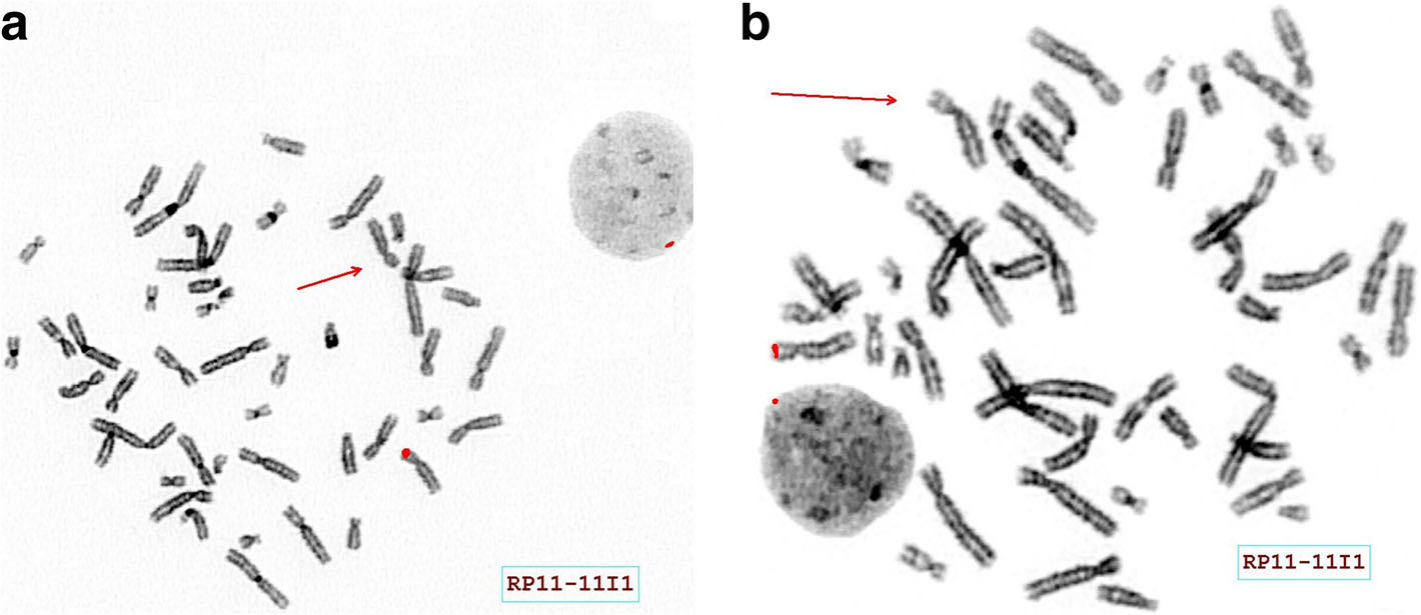
FISH analysis. FISH analysis using the BlueGnome FISH probe RP11-11I1 show heterozygous deletion (one red signal) in his mother (**a**) and father (**b**). The chromosomes with deletions are indicated by an arrow

The homozygous deletion detected in our proband was also observed in one of his brothers and segregated with microcephaly and intellectual disability (Fig. 3). The parents were both found to be hemizygous for this deletion (Fig. 3) and admitted to consanguinity, which supported the autosomal recessive nature of this pathogenic deletion for microcephaly and intellectual disability. FISH testing confirmed homozygous deletion of the *MCPH1* gene in the proband and hemizygous deletion of the same gene in his mother and father (Fig. 4a, b). The older, unaffected brother was not available for testing.

The clinical and molecular features of the affected siblings presented herein are similar to those of patients reported in the literature to also have *MCPH1* deletions (Table 1) [11, 12]. Garshasbi et al. reported 6 individuals (4 males, 2 females) of an Iranian family who had primary microencaphly had a 150-200 kb deletion (8p22.2p23.2), which spanned 25 kb of the promoter sequence of *MCPH1* and exons 1-6 [11]. Microcephaly in these individuals was borderline to moderate and mental retardation was mild to moderate [11]. A single case report of primary microencaphly caused by a microdeletion exists of a male Asian Indian 2 month-old baby [12]. For this subject, the deletion (8p23.1(6197,889-6295,040)×1) involved exons 1-11 of *MCPH1* [12]. From these few reports it appears that greater genomic alteration of the *MCPH1* gene may be indicative of more significant clinical phenotypes, since studies of patients with point mutations in *MCPH1* suggest milder phenotypes [13].

**Table 1:** Clinical and Molecular Presentation of Proband and Comparison to Patients Reported in the Literature to have *MCPH1* Deletions

Features	Proband, 1 Patient	Garshasbi 2006 [11], 6 Patients	Pfau 2013 [12], 1 Patient
Age	35 months	18-32 years	10 months
Gender	Male	4 Males, 2 females	Male
Race	Hispanic	Iranian	Asian Indian
Microcephaly (Head circumference)	44 cm (<−2 SD)	49-50 cm (−3 SD), borderline to mild microcephaly	38 cm (<−5 SD)
Chromosome deletion	8p23.2p23.1 (hg19: 6,061,169-6,310,738)	8p22.2p23.2 (hg19: ~6239 kb-6300 kb)	8p23.1(hg19: 6164,466 × 2,6197,889 × 1-6295,040 × 1,6339,527 × 2)
Deletion description	250 kb (203 kb of upstream sequence and exons 1-8 of 14 (NM_024596)	150-200 kb that covered 25 kb of upstream sequence and exons 1-6 of 11	97-175 kb, promoter region and exons 1-11 of 14
Facial features	Almond shaped eyes, epicanthal folds, bilateral esotropia, low hairline, large ears, smooth philtrum, and thin upper lip	Not described	Mild facial dysmorphism
Intellectual disability	Physical and speech delay. At 48 months, <25% of his speech was intelligible to strangers, and he did not form sentences.	Mild to moderate mental retardation	Developmentally, patient had a social smile, stranger recognition, verbalized with coos and babbling, clapped when asked, and was able to progress from lying recumbent to rolling over, sitting and crawling without assistance. His progress had resulted in graduation from state developmental services.

**Fig. 5:**
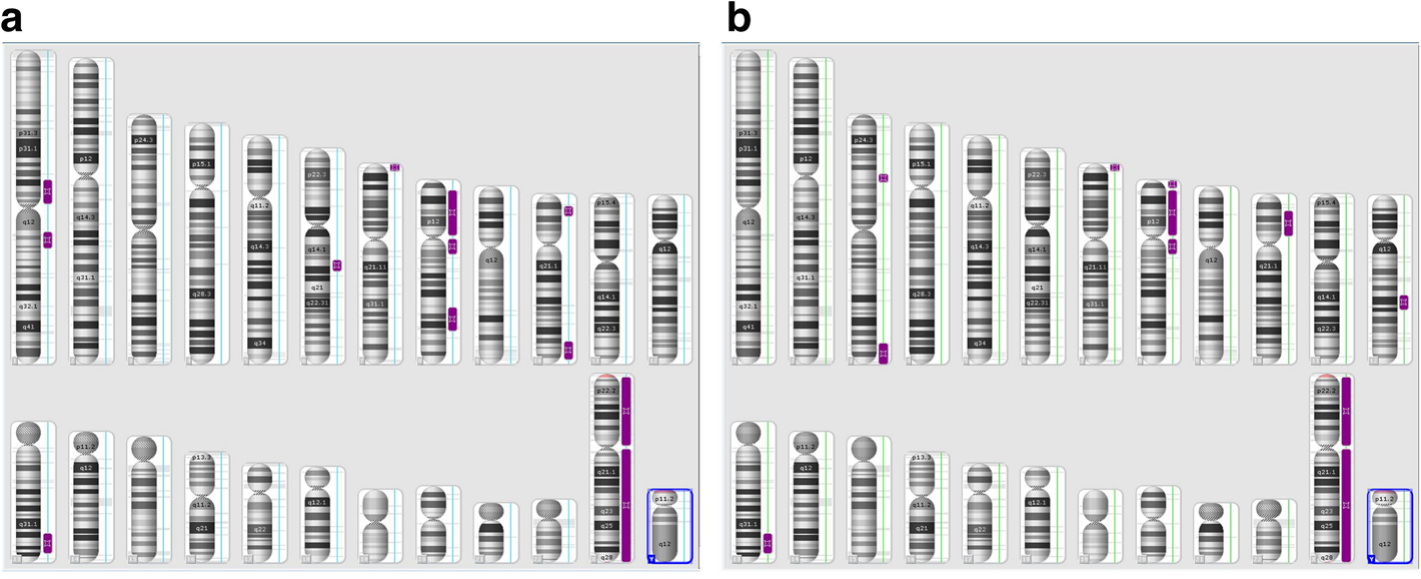
SNP-array results showing loss of heterozygosity (ROH) in the proband (**a**) and his brother (**b**). The purple bars next to the corresponding chromosomes indicate ROHs

In addition to homozygous copy number loss, many copy neutral regions of homozygosity (ROHs) were observed in both the patient (Fig. 5a) and his brother (Fig. 5b). ROHs that span >5 Mb totaled approximately 148 Mb across the genome. The implications of ROHs are unclear at present. In theory, when the degree of homozygosity is significant, there is an increased risk for recessive Mendelian disorders. Since the clinical features of our patient and the previously reported cases with exonic deletions are similar, the ROHs found in our patient are likely to have no or minimum impact on the patient’s phenotype.

## Conclusions

Here we report a homozygous deletion of multiple exons of the *MCPH1* gene that was associated with primary microcephaly and intellectual disability; to our knowledge, this is the first such report in a Hispanic family. In the context of previous studies, our results support the idea that deletions involving multiple exons cause a more severe phenotype than point mutations.

## Abbreviations

CMA: Chromosomal microarray analysis
MCPH1: Microcephalin gene
Oligo-SNP array: Oligonucleotide-single nucleotide polymorphism array
